# Effects of Elevated Temperature on *Pisum sativum* Nodule Development: II—Phytohormonal Responses

**DOI:** 10.3390/ijms242317062

**Published:** 2023-12-02

**Authors:** Anna B. Kitaeva, Tatiana A. Serova, Pyotr G. Kusakin, Viktor E. Tsyganov

**Affiliations:** Laboratory of Molecular and Cell Biology, All-Russia Research Institute for Agricultural Microbiology, 196608 Saint Petersburg, Russia; t.serova@arriam.ru (T.A.S.); pyotr.kusakin@arriam.ru (P.G.K.)

**Keywords:** abiotic stress, heat stress, legume–rhizobial symbiosis, symbiotic nodule, abscisic acid, ethylene, gibberellins

## Abstract

High temperature is one of the most important factors limiting legume productivity. We have previously shown the induction of senescence in the apical part of nodules of the pea SGE line, formed by *Rhizobium leguminosarum* bv. *viciae* strain 3841, when they were exposed to elevated temperature (28 °C). In this study, we analyzed the potential involvement of abscisic acid (ABA), ethylene, and gibberellins in apical senescence in pea nodules under elevated temperature. Immunolocalization revealed an increase in ABA and 1-aminocyclopropane-1-carboxylic acid (ACC, the precursor of ethylene biosynthesis) levels in cells of the nitrogen fixation zone in heat-stressed nodules in 1 day of exposure compared to heat-unstressed nodules. Both ABA and ethylene appear to be involved in the earliest responses of nodules to heat stress. A decrease in the gibberellic acid (GA_3_) level in heat-stressed nodules was observed. Exogenous GA_3_ treatment induced a delay in the degradation of the nitrogen fixation zone in heat-stressed nodules. At the same time, a decrease in the expression level of many genes associated with nodule senescence, heat shock, and defense responses in pea nodules treated with GA_3_ at an elevated temperature was detected. Therefore, apical senescence in heat-stressed nodules is regulated by phytohormones in a manner similar to natural senescence. Gibberellins can be considered as negative regulators, while ABA and ethylene can be considered positive regulators.

## 1. Introduction

High temperature is one of the main factors affecting yield and seed quality in legumes [1,2]. Heat stress for plants begins when the air temperature increases at least one degree above a threshold level [3]. The upper threshold temperature is referred to as the temperature at which seed germination, seedling and vegetative development, flowering, fruit set, and fruit ripening begin to be seriously affected [4].

Pea (*Pisum sativum* L.) is a cool-season food legume [5], and for its optimal growth, the mean seasonal temperature should not exceed 18 °C [6]. Cool-season food legumes are considered more sensitive to heat stress compared with warm-season food legumes [7,8]. Among other cool-season food legumes such as chickpea (*Cicer arietinum* L.) and lentil (*Lens culinaris* Medik.), pea is characterized by lower heat stress tolerance [9]; the minimum temperature for peas is 15 °C, while the maximum temperature is 25 °C [10]. However, Fletcher and co-authors considered 20–21 °C to be the optimal temperature [11]. There are several reports that pea productivity decreases significantly when the maximum air temperature exceeds 25 °C [12,13,14]. Other researchers reported that 32 °C [15], 27 °C [16], or 36 °C [17] were critical for pea yield reduction. It is supposed that the effect of heat stress varies depending on the temperature and duration of exposure [4]. In peas, high temperature influences a number of physiological parameters like photosynthetic rate [18], nitrogen fixation rate [19], nitrogen assimilate remobilization [20], seed germination [21], and the duration of reproductive phase [22].

An important feature of pea, as well as other legumes, is the ability to form symbiotic nodules. Pea forms nodules of indeterminate type, which are characterized by the presence of several histological zones resulting from prolonged meristem activity [23]. These zones are meristem, infection zone, nitrogen fixation zone, and the senescence zone, which appears with age at the base of the nodule [24]. Nodule development is under complex regulation, including phytohormonal regulation [25,26]. The role of various phytohormones, such as ethylene [27,28], gibberellins [29,30], cytokinins and auxins [31,32,33,34,35,36], and strigolactones [37,38,39], in nodule development in pea has been demonstrated.

It should be noted that studies on the effect of heat stress on the development and functioning of pea symbiotic nodules are extremely limited. Recently, in our study of the symbiosis formed by pea plants of the laboratory line SGE with *Rhizobium leguminosarum* bv. *viciae* strain 3841, we observed an unusual senescence pattern in the apical part of nodules induced at elevated temperature [40]. The induction of senescence in the apical part of the nodule has never been previously described for pea nodules exposed to different stressors [41,42,43] nor during natural senescence. It is well-known that natural nodule senescence is controlled by various factors, including phytohormones [44,45,46,47]. A detailed analysis of pea nodule senescence has revealed that gibberellins are positive regulators of senescence, while ethylene and abscisic acid (ABA) are negative regulators [48,49].

The aim of this study was to investigate the potential involvement of ethylene, ABA, and gibberellins in the induction of senescence in the apical part of pea nodules exposed to heat stress. For this purpose, immunolocalization of phytohormones, pharmacological approach (treatment with aminoethoxyvinylglycine hydrochloride (AVG, ethylene biosynthesis inhibitor)) and gibberellic acid (GA_3_)), light microscopy, and expression analysis of marker genes associated with nodule senescence, heat shock, and defense responses were used.

## 2. Results

### 2.1. ABA Immunolocalization

**Figure 1:**
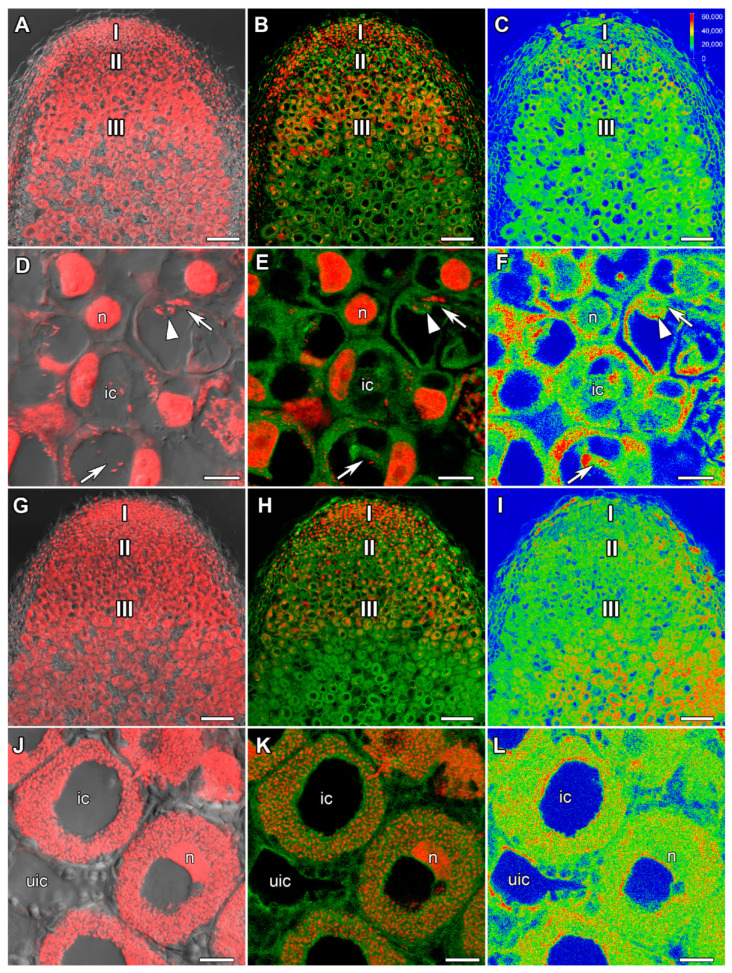
Immunolocalization of abscisic acid (ABA) in 22-day-old nodules of the pea (*Pisum sativum*) line SGE. (**A**–**F**) Heat-unstressed nodules; (**G**–**L**) heat-stressed nodules. (**A**–**C**,**G**–**I**) Whole nodules; (**D**–**F**) early infection zone; (**J**–**L**) nitrogen fixation zone. Confocal laser scanning microscopy of 50 μm longitudinal vibratome sections. (**A**,**D**,**G**,**J**) Merge of the differential interference contrast and the red channel (DNA staining with propidium iodide (nuclei and bacteria)). (**B**,**E**,**H**,**K**) Merge of green (ABA) and red (propidium iodide) channels. (**C**,**F**,**I**,**L**) The heatmap shows color-coded fluorescence signal intensities for the green signal channel; the quantification scale is the same for all images. I, meristem; II, infection zone; III, nitrogen fixation zone. ic, infected cell; uic, uninfected cell; n, nucleus; arrows indicate infection threads; arrowheads indicate infection droplets. Scale bars are 100 μm (**A**–**C**,**G**–**I**) and 10 μm (**D**–**F**,**J**–**L**).

In 22-day-old heat-unstressed nodules, the maximal intensity of ABA was detected in cells of the early infection zone, i.e., in recently infected cells (Figure 1A–C). The label was mainly localized in the cytoplasm (Figure 1D–F). In 1 day of exposure to elevated temperature (28 °C), an increase in the amount of the label in heat-stressed nodules compared to heat-unstressed ones was observed. The maximal amount of the label was observed in the nitrogen fixation zone (Figure 1G–I). ABA was localized in the cytoplasm of both infected and uninfected cells. Some amount of the label was observed in the nuclei of infected cells (Figure 1J–L).

**Figure 2:**
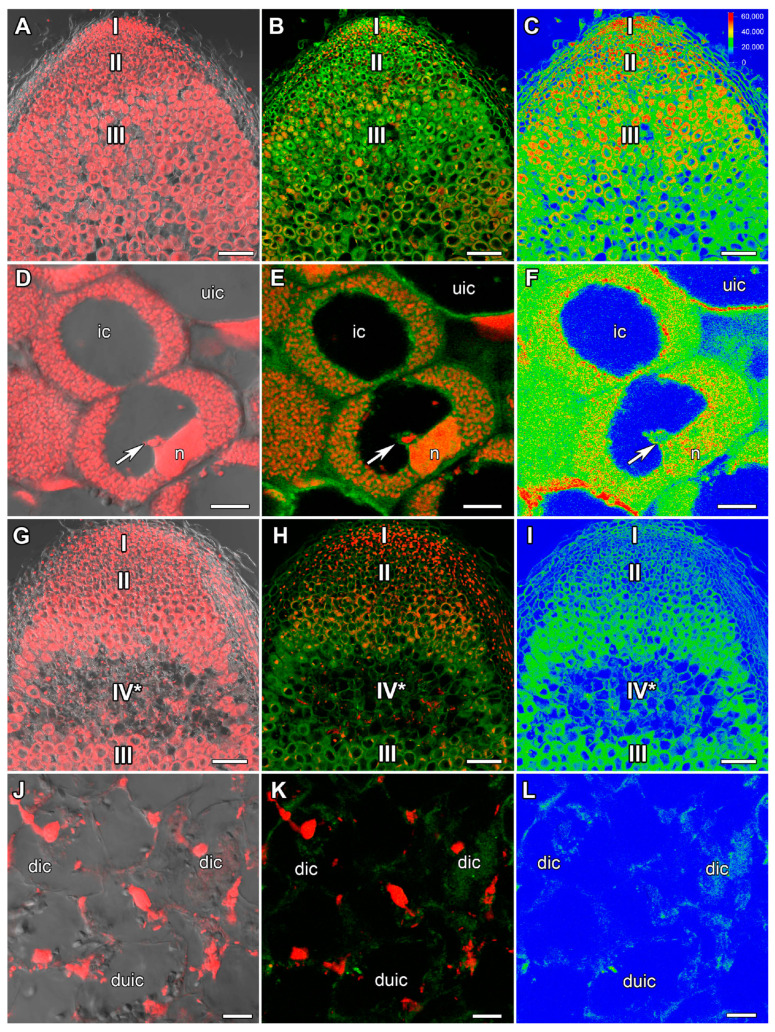
Immunolocalization of abscisic acid (ABA) in 26-day-old nodules of the pea (*Pisum sativum*) line SGE. (**A**–**F**) Heat-unstressed nodules; (**G**–**L**) heat-stressed nodules. (**A**–**C**,**G**–**I**) Whole nodules; (**D**–**F**) nitrogen fixation zone; (**J**–**L**) senescence zone in the apical part of the nodule. Confocal laser scanning microscopy of 50 μm longitudinal vibratome sections. (**A**,**D**,**G**,**J**) Merge of the differential interference contrast and the red channel (DNA staining with propidium iodide (nuclei and bacteria)). (**B**,**E**,**H**,**K**) Merge of green (ABA) and red (propidium iodide) channels. (**C**,**F**,**I**,**L**) The heatmap shows color-coded fluorescence signal intensities for the green signal channel; the quantification scale is the same for all images. I, meristem; II, infection zone; III, nitrogen fixation zone; IV*, senescence zone at the apical part of the nodule. ic, infected cell; uic, uninfected cell; dic, degraded infected cell; duic, degraded uninfected cell; n, nucleus; arrows indicate infection threads. Scale bars are 100 μm (**A**–**C**,**G**–**I**) and 10 μm (**D**–**F**,**J**–**L**).

In 26-day-old heat-unstressed nodules, the amount of the ABA label was increased (Figure 2A–C) compared with 22-day-old heat-unstressed nodules. The maximal amount of the label was localized in the meristem, the infection zone, and the distal part of the nitrogen fixation zone (Figure 2A–F). In nodules heat-stressed for 5 days, a sharp decrease in ABA amount (Figure 2G–I) compared with heat-unstressed nodules was observed. In the senescence zone in the apical part of the nodule, the label was localized in degraded infected and uninfected cells (Figure 2J–L).

In 30-day-old heat-unstressed nodules, the maximal amount of the label was observed in the nitrogen fixation zone (Figure S1A–C). The signal was observed in the cytoplasm and nuclei of infected and uninfected cells (Figure S1D–F). In nodules heat-stressed for 9 days, the intensity of the ABA label noticeably decreased and was found in degraded cells (Figure S1G–L).

### 2.2. ACC Immunolocalization

**Figure 3:**
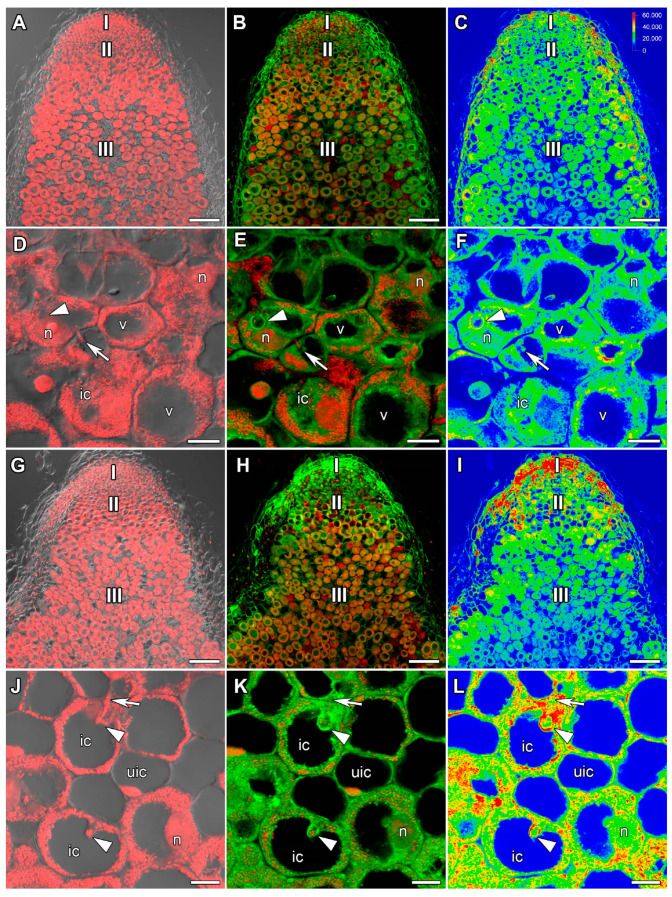
Immunolocalization of 1-aminocyclopropane-1-carboxylate (ACC) in 22-day-old nodules of the pea (*Pisum sativum*) line SGE. (**A**–**F**) heat-unstressed nodules; (**G**–**L**) heat-stressed nodules. (**A**–**C**,**G**–**I**) Whole nodules; (**D**–**F**,**J**–**L**) infection zone. Confocal laser scanning microscopy of 50 μm longitudinal vibratome sections. (**A**,**D**,**G**,**J**) Merge of the differential interference contrast and the red channel (DNA staining with propidium iodide (nuclei and bacteria)). (**B**,**E**,**H**,**K**) Merge of green (ACC) and red (propidium iodide) channels. (**C**,**F**,**I**,**L**) The heatmap shows color-coded fluorescence signal intensities for the green signal channel; the quantification scale is the same for all images. I, meristem; II, infection zone; III, nitrogen fixation zone. ic, infected cell; uic, uninfected cell; n, nucleus; v, vacuole; arrows indicate infection threads; arrowheads indicate infection droplets. Scale bars are 100 μm (**A**–**C**,**G**–**I**) and 10 μm (**D**–**F**,**J**–**L**).

In 22-day-old heat-unstressed nodules, the ACC label was evenly distributed in all nodule zones besides peripheral tissues (Figure 3A–C). However, some increase in ACC amount was noticed in the cytoplasm around vacuoles and infection droplets in cells of the infection zone (Figure 3D–F). In nodules heat-stressed for 1 day, the level of ACC was higher compared with heat-unstressed nodules. The maximal amount was observed in the meristem and in the infection zone (Figure 3G–I). In the infection zone, the maximal level of ACC was localized in the cytoplasm, nuclei, and around infection droplets (Figure 3J–L).

**Figure 4:**
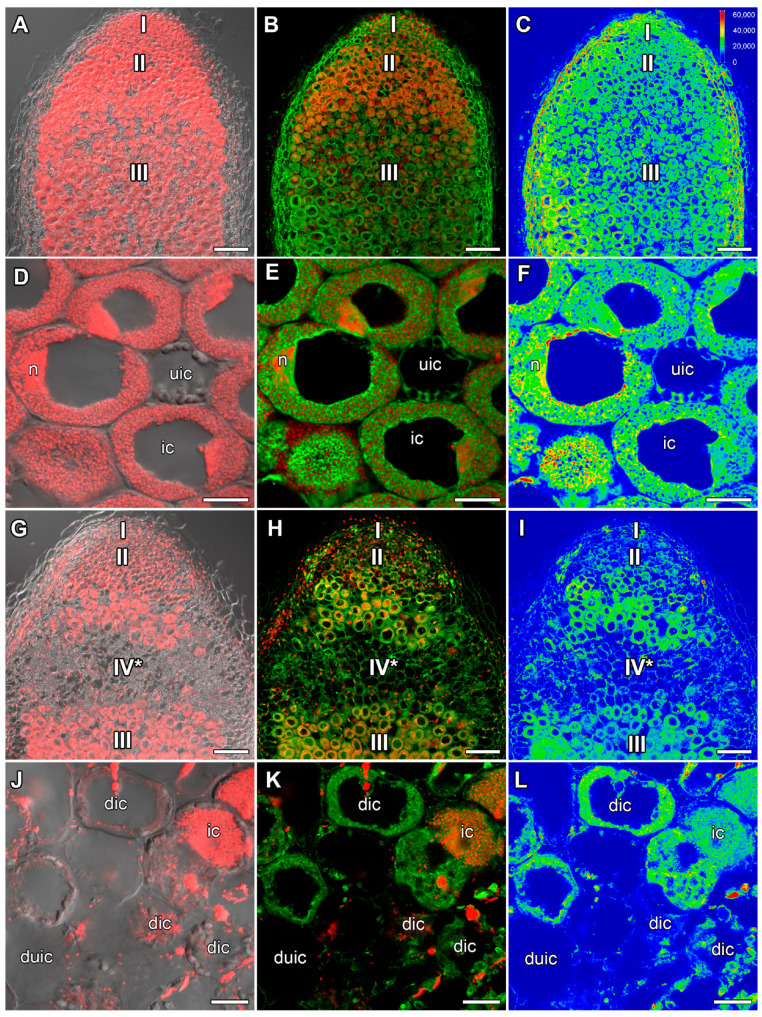
Immunolocalization of 1-aminocyclopropane-1-carboxylate (ACC) in 26-day-old nodules of the pea (*Pisum sativum*) line SGE. (**A**–**F**) heat-unstressed nodules; (**G**–**L**) heat-stressed nodules. (**A**–**C**,**G**–**I**) Whole nodules; (**D**–**F**) nitrogen fixation zone; (**J**–**L**) senescence zone in the apical part of the nodule. Confocal laser scanning microscopy of 50 μm longitudinal vibratome sections. (**A**,**D**,**G**,**J**) Merge of the differential interference contrast and the red channel (DNA staining with propidium iodide (nuclei and bacteria)). (**B**,**E**,**H**,**K**) Merge of green (ACC) and red (propidium iodide) channels. (**C**,**F**,**I**,**L**) The heatmap shows color-coded fluorescence signal intensities for the green signal channel; the quantification scale is the same for all images. I, meristem; II, infection zone; III, nitrogen fixation zone; IV*, senescence zone at the apical part of the nodule. ic, infected cell; uic, uninfected cell; dic, degraded infected cell; duic, degraded uninfected cell; n, nucleus. Scale bars are 100 μm (**A**–**C**,**G**–**I**) and 20 μm (**D**–**F**,**J**–**L**).

In 26-day-old heat-unstressed nodules, the amount of ACC was similar to that in 22-day-old heat-unstressed nodules (Figure 4A–C). The maximal amount of the label was observed around vacuoles in infected cells (Figure 4D–F). In 5 days of exposure to elevated temperature, a significant decrease in ACC intensity was observed compared with nodules that were heat-stressed for 1 day (Figure 4G–I). The ACC label was localized in cells of the late infection zone and in the nitrogen fixation zone. Also, the ACC label was detected in degraded cells in the senescence zone in the apical part of the nodule (Figure 4J–L).

In 30-day-old heat-unstressed nodules, the maximal amount of the ACC label was localized in cortical and meristematic cells (Figure S2A–C). In the infected cells, the ACC label was distributed throughout the cytoplasm and nuclei (Figure S2D–F). In nodules heat-stressed for 9 days, the ACC label was localized in the nuclei and cytoplasm in nodule cortical cells. In addition, high intensity of the ACC label was revealed in degraded cells (Figure S2G–L).

### 2.3. GA_3_ Immunolocalization

**Figure 5:**
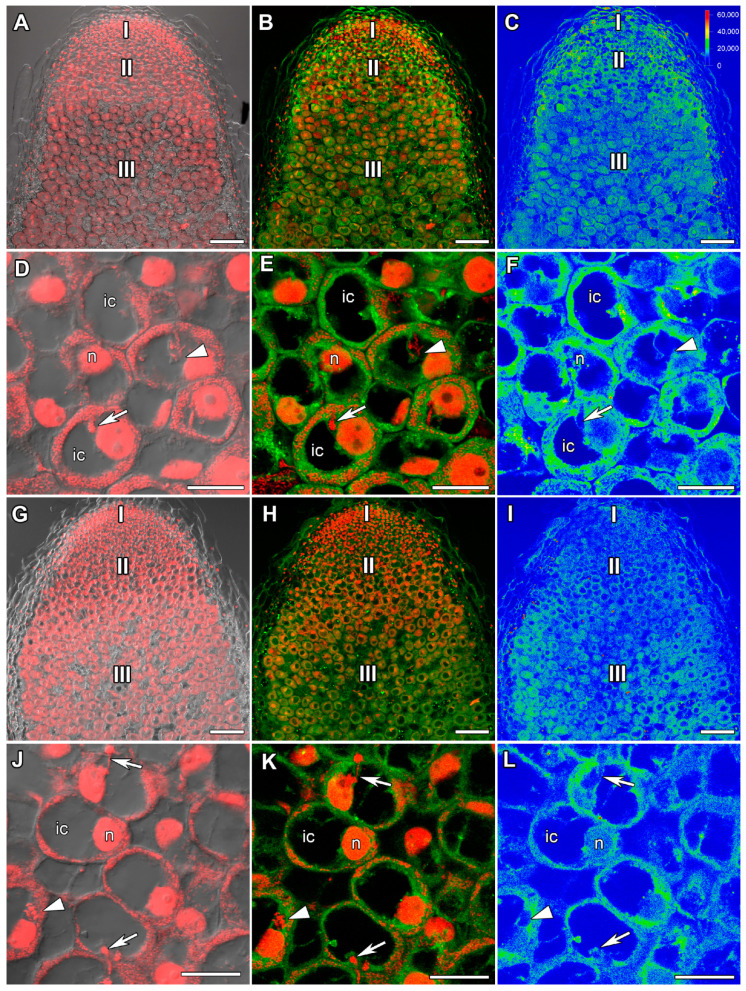
Immunolocalization of gibberellic acid (GA_3_) in 22-day-old nodules of the pea (*Pisum sativum*) line SGE. (**A**–**F**) heat-unstressed nodules; (**G**–**L**) heat-stressed nodules. (**A**–**C**,**G**–**I**) Whole nodules; (**D**–**F**,**J**–**L**) infection zone. Confocal laser scanning microscopy of 50 μm longitudinal vibratome sections. (**A**,**D**,**G**,**J**) Merge of the differential interference contrast and the red channel (DNA staining with propidium iodide (nuclei and bacteria)). (**B**,**E**,**H**,**K**) Merge of green (GA_3_) and red (propidium iodide) channels. (**C**,**F**,**I**,**L**) The heatmap shows color-coded fluorescence signal intensities for the green signal channel; the quantification scale is the same for all images. I, meristem; II, infection zone; III, nitrogen fixation zone. ic, infected cell; n, nucleus; arrows indicate infection threads; arrowheads indicate infection droplets. Scale bars are 100 μm (**A**–**C**,**G**–**I**) and 20 μm (**D**–**F**,**J**–**L**).

In 22-day-old heat-unstressed nodules, the GA_3_ label was distributed throughout all nodule zones with an increase in intensity in the meristem and the infection zone (Figure 5A–C). In cells of the infection zone, the GA_3_ label was localized in the cytoplasm and partially in nuclei (Figure 5D–F). In nodules heat-stressed for 1 day, some decrease in GA_3_ amount in the meristem and infection zone was observed (Figure 5G–I). A relatively high level of label intensity was detected in the cytoplasm (Figure 5J–L).

**Figure 6:**
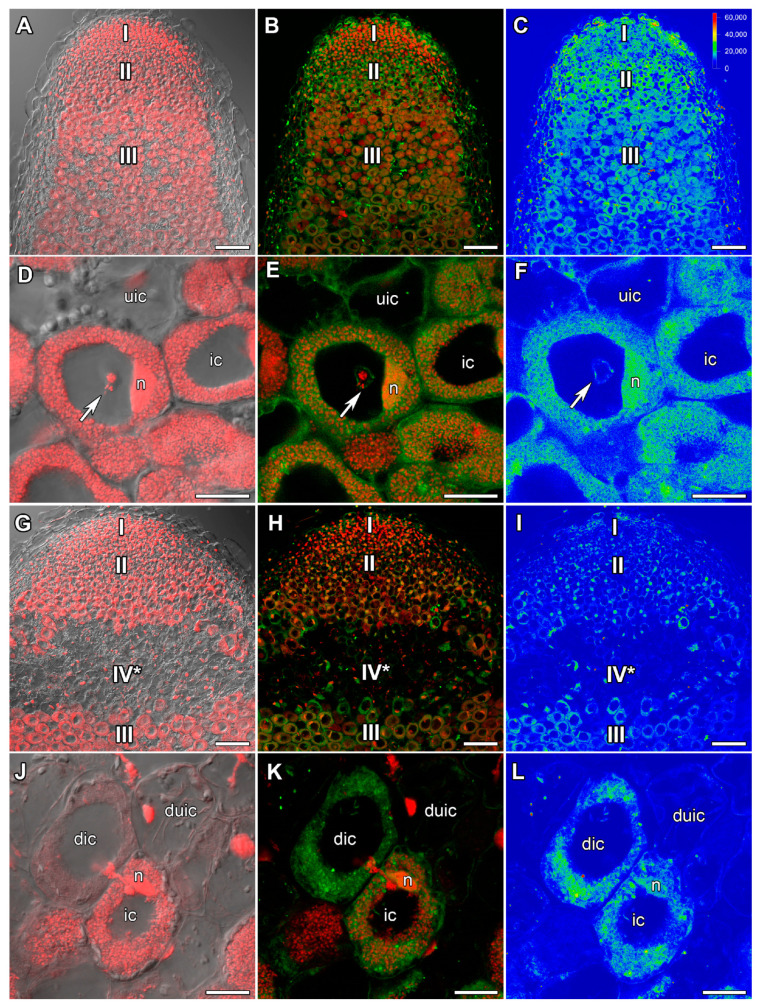
Immunolocalization of gibberellic acid (GA_3_) in 26-day-old nodules of the pea (*Pisum sativum*) line SGE. (**A**–**F**) heat-unstressed nodules; (**G**–**L**) heat-stressed nodules. (**A**–**C**,**G**–**I**) Whole nodules; (**D**–**F**) nitrogen fixation zone; (**J**–**L**) cells adjacent to the senescence zone in the apical part of the nodule. Confocal laser scanning microscopy of 50 μm longitudinal vibratome sections. (**A**,**D**,**G**,**J**) Merge of the differential interference contrast and the red channel (DNA staining with propidium iodide (nuclei and bacteria)). (**B**, **E**,**H**,**K**) Merge of green (GA_3_) and red (propidium iodide) channels. (**C**,**F**,**I**,**L**) The heatmap shows color-coded fluorescence signal intensities for the green signal channel; the quantification scale is the same for all images. I, meristem; II, infection zone; III, nitrogen fixation zone; IV*, senescence zone at the apical part of the nodule. ic, infected cell; uic, uninfected cell; dic, degraded infected cell; duic, degraded uninfected cell; n, nucleus; arrows indicate infection threads. Scale bars are 100 μm (**A**–**C**,**G**–**I**) and 20 μm (**D**–**F**,**J**–**L**).

In 26-day-old heat-unstressed nodules, the amount of GA_3_ was similar to that in 22-day-old heat-unstressed nodules (Figure 6A–F). In 5 days of exposure to elevated temperature, GA_3_ amount in heat-stressed nodules was significantly decreased (Figure 6G–I). The GA_3_ label was predominantly localized in the nuclei in the infection and nitrogen fixation zones. In addition, the label was found in infected cells at the early stage of degradation and adjacent to the senescence zone in the apical part of the nodule (Figure 6J–L).

In 30-day-old heat-unstressed nodules, the GA_3_ amount was slightly decreased compared with 26-day-old nodules (Figure S3A–F). In nodules heat-stressed for 9 days, the GA_3_ label was almost absent (Figure S3G–I). Only a very weak signal was detected in some degraded cells (Figure S3J–L).

### 2.4. Nodule Phenotype in Pea Plants of the Line SGE Exposed to Elevated Temperature in Combination with Pharmacological Treatment

**Figure 7:**
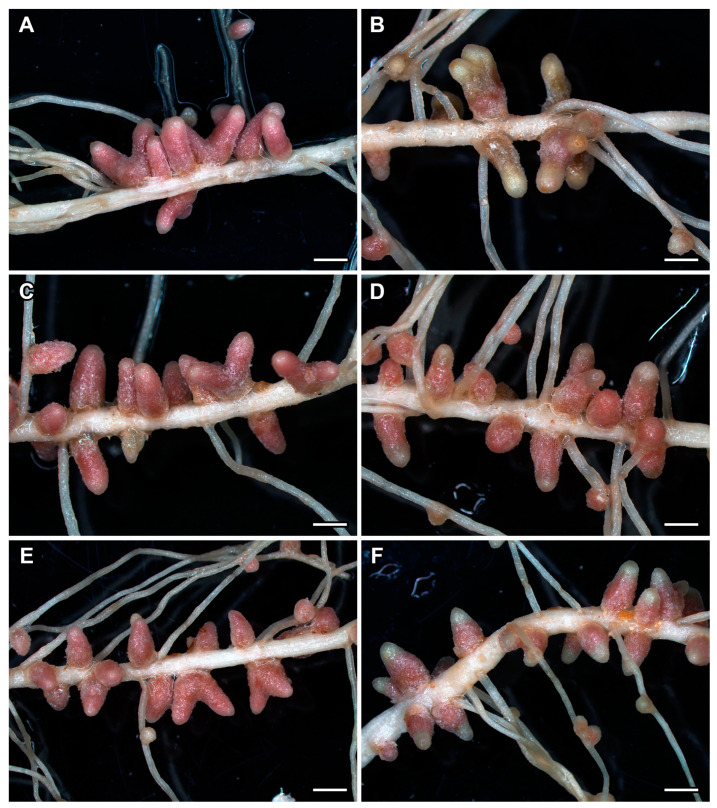
Heat-unstressed (**A**,**C**,**E**) and heat-stressed (**B**,**D**,**F**) 28-day-old nodules of the pea (*Pisum sativum*) line SGE untreated (**A**,**B**) and treated with 1 μM gibberellic acid (GA_3_) (**C**,**D**) or 100 μM aminoethoxyvinylglycine hydrochloride (AVG) (**E**,**F**). Scale bars are 2 mm.

In 28- and 30-day-old heat-unstressed plants, typical pink nodules were observed both without treatment (Figure 7A and Figure S4A) and in combination with pharmacological treatment (GA_3_ or ethylene biosynthesis inhibitor, AVG) (Figure 7C,E and Figure S4C,E). In nodules of plants heat-stressed for 7 and 9 days, a color change from pink to light green was noted in their apical part and a change from pink to brownish in their bases (Figure 7B and Figure S4B).

In GA_3_-treated plants heat-stressed for 7 and 9 days, nodules with a green apical part were observed; however, in such nodules, a large part of the nodule retained pink coloration (Figure 7D and Figure S4D) in contrast to GA_3_-untreated plants. Nodules of GA_3_-treated plants heat-stressed for 9 days had a more pronounced green coloration of their apical parts (Figure S4D).

The nodules of AVG-treated plants heat-stressed for 7 days were characterized by a more intense green coloration of their apical parts (Figure 7F) compared to the nodules of GA_3_-treated heat-stressed plants (Figure 7D). At the same time, in such nodules, a large part of the nodule retained pink coloration (Figure 7F) in contrast to the untreated nodules of heat-stressed plants (Figure 7B). In AVG-treated plants heat-stressed for 9 days, a significant expansion of green coloration was observed from the apical part of the nodule to its base (Figure S4F).

### 2.5. Histological Organization of Nodules in Pea Plants of the Line SGE Exposed to Elevated Temperature in Combination with Pharmacological Treatment

**Figure 8:**
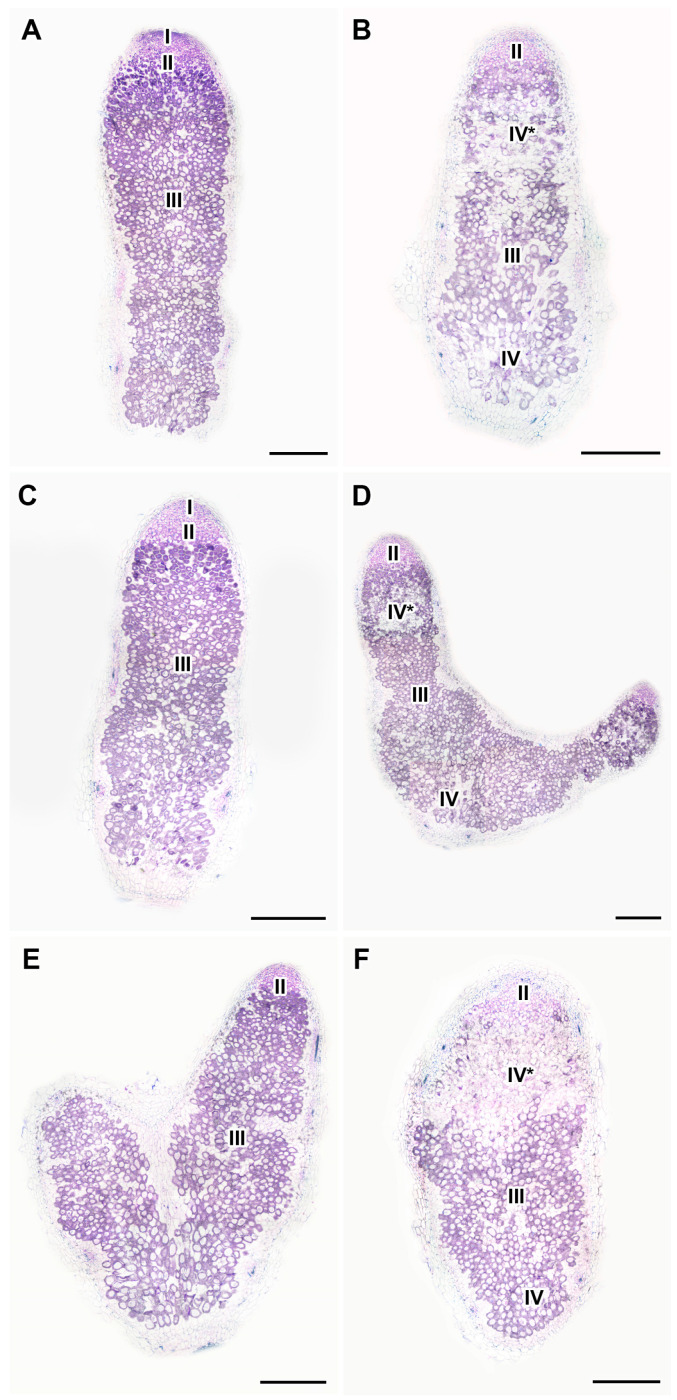
Histological organization of heat-unstressed (**A**,**C**,**E**) and heat-stressed (**B**,**D**,**F**) 28-day-old nodules of the pea (*Pisum sativum*) line SGE untreated (**A**,**B**) and treated with 1 μM gibberellic acid (GA_3_) (**C**,**D**) or 100 μM aminoethoxyvinylglycine hydrochloride (AVG) (**E**,**F**). Light microscopy of 10 μm longitudinal microtome sections, stained with toluidine blue. I, meristem; II, infection zone; III, nitrogen fixation zone; IV, senescence zone; IV*, senescence zone at the apical part of the nodule. Scale bars are 500 µm.

In nodules of 28- and 30-day-old heat-unstressed untreated plants, several histological zones, including the meristem, infection zone, and nitrogen fixation zone were observed (Figure 8A and Figure S5A). A similar organization of nodules was observed in heat-unstressed plants treated with GA_3_ or AVG (Figure 8C,E and Figure S5C,E).

In nodules of untreated plants heat-stressed for 7 days, the senescence zone appeared both in the apical part of the nodule (Figure 8B) and at its base (Figure 8B). In plants heat-stressed for 9 days, the senescence zone occupied a significant part of the nodule (Figure S5B).

In plants heat-stressed for 7 days with simultaneous GA_3_ treatment, the senescence zone in the apical part of the nodule was less pronounced (Figure 8D), and only single degraded cells were observed in its basal part (Figure 8D). In plants heat-stressed for 9 days, an increase in the area of tissue degradation in the apical part of the nodule was observed (Figure S5D), while the nitrogen fixation zone was still pronounced (Figure S5D).

In AVG-treated plants heat-stressed for 7 days, a significant degradation of the cells in the apical part of the nodule was observed (Figure 8F). In plants heat-stressed for 9 days, a pronounced degradation of the cells of the whole nodule was observed; however, a small nitrogen fixation zone was distinguished (Figure S5F).

### 2.6. Expression Analysis of the Genes Associated with Senescence, Heat Stress, and Defense Responses in Nodules of Pea Plants of the Line SGE Exposed to Elevated Temperature in Combination with GA_3_ Treatment

**Figure 9:**
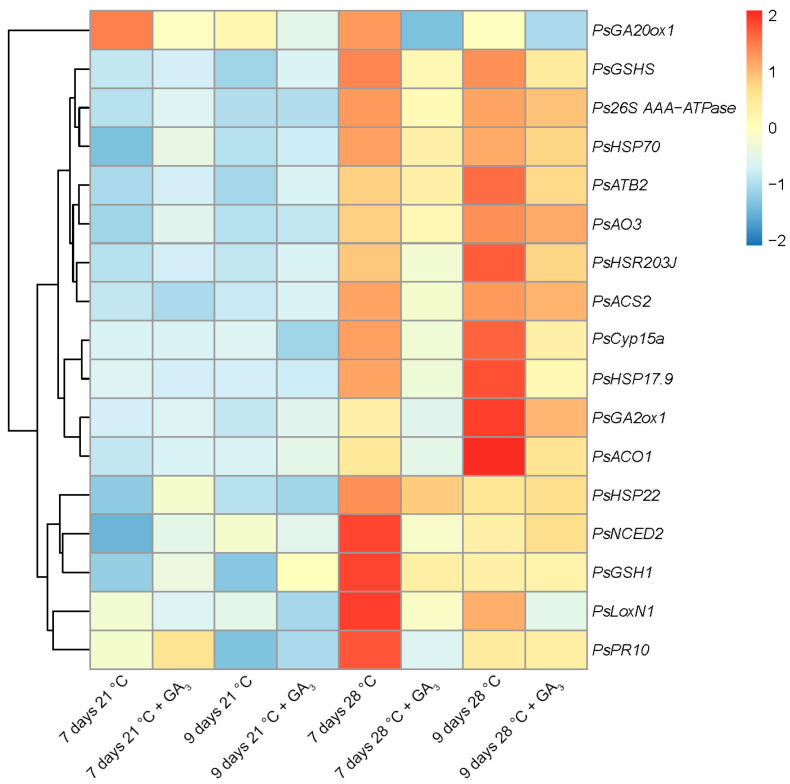
Heatmap showing relative gene expression levels in nodules of pea (*Pisum sativum*) plants of the SGE line exposed to 21 °C or 28 °C for 7 or 9 days with or without gibberellic acid (GA_3_) treatment. Transcript levels were determined by real-time PCR and calculated using the ΔC_t_ method with glyceraldehyde-3-phosphate dehydrogenase (*PsGapC1*) serving as the reference gene. The color scale shows relative expression values for each gene after Z-transformation. The heatmap is based on data presented in Table S1 (list 1). Gene expression levels were compared using one-way ANOVA. A *p*-value < 0.05 was considered significant (see also Table S1 (list 2)).

The expression levels of genes associated with senescence (cysteine protease 15a (*PsCyp15a*), P45 subunit of the AAA-ATPase 26S proteasome (*Ps26S AAA-ATPase*), transcription factor bZIP (*PsATB2*), enzymes for the GA metabolism (*PsGA20ox1*, *PsGA2ox1*), ethylene biosynthesis (*PsACS2*, *PsACO1*), ABA biosynthesis (*PsNCED2*, *PsAO3*)), heat stress (heat shock proteins (*PsHSP22*, *PsHSP17.9*, *PsHSP70*)) and defense responses (biosynthesis of jasmonates (*PsLoxN1*) and glutathione (*PsGSH1*, *PsGSHS*), the marker of hypersensitivity reaction (*PsHSR203J*), and disease resistance response protein (*PsPR10*)) were analyzed in nodules of GA_3_-treated and untreated plants heat-stressed and heat-unstressed for 7 and 9 days (Figure 9).

The highest expression levels for most of the analyzed genes were observed in nodules of untreated heat-stressed plants (Figure 9). In nodules of heat-unstressed plants with and without GA_3_ treatment, the transcript abundance was significantly lower than in heat-stressed nodules (with the exception of *PsGA20ox1*, the GA_3_ biosynthesis enzyme). In GA_3_-treated nodules heat-stressed for 7 days, a decrease in the expression level of all analyzed genes was shown (Figure 9). In GA_3_-treated nodules heat-stressed for 9 days, similar changes in the transcript abundance were significant only for the part of analyzed genes (Figure 9). This observation confirms the increase in the senescence zone in GA_3_-treated nodules heat-stressed for 9 days compared to those heat-stressed for 7 days (Figure 8D and Figure S5D).

## 3. Discussion

At different developmental stages, the growth of plants is affected by abiotic stresses like temperature, drought, and salt [50]. Abiotic stresses are considered a major cause of yield loss worldwide, resulting in >50% yield reductions in most plants [51]. Heat stress disrupts various physiological processes such as photosynthesis, carbon fixation and assimilation [52], impairs electron transport [53], causes leaf dehydration [8], inhibition of various enzymes [54], alterations in antioxidant defense systems [55], and damage to vegetative and reproductive development [56], and negatively influences nodule development and nitrogen fixation [57].

In our previous study, we found the activation of apical senescence in heat-stressed pea nodules of the SGE line formed by the *Rhizobium leguminosarum* bv. *viciae* strain 3841 [40]. Detailed characterization of this phenomenon showed its similarity at the transcriptional and cellular levels with natural senescence in the basal part of the nodule. It is known that phytohormones are involved in the control of natural and induced nodule senescence [44,45,48,49,58,59,60,61,62]. In this study, we examined the involvement of ABA, ethylene, and GAs in the process of apical senescence induced by temperature stress.

In this study, an increase in the amount of the ABA label in cells of the nitrogen fixation zone in 1 day of exposure to elevated temperature compared with heat-unstressed nodules was revealed (Figure 1). With further exposure to elevated temperature, the level of ABA in nodules decreased markedly (Figure 2 and Figure S1). Previously, increased expression of ABA biosynthesis genes was shown in the natural senescence of common bean (*Phaseolus vulgaris* L.) [63] and pea nodules [48,58], as well as senescence induced by mutations in pea symbiotic genes [48,58] and heat stress [40]. It is evident that the increase in the ABA level in the nodule is one of the early plant responses to heat stress, the intensity of which decreases with time. Interestingly, after 24 h of heat stress at 38 °C, the highest level of ABA accumulation was found in pea stipuli [64]. In addition, an increase in the expression of one of the ABA biosynthesis genes *ZEP* (*ZEAXANTHIN EPOXIDASE*) and ABA content was observed in *Lupinus luteus* L. nodules exposed to drought, which is likely related to the induction of senescence in such nodules [65].

An increase in the intensity of the ACC label in cells from the meristem and infection zone in nodules heat-stressed for 1 day compared with heat-unstressed nodules was observed (Figure 3). It should be noted that with further exposure, the level of ACC decreased, but a high level of ACC was maintained in degraded cells of nodules heat-stressed for 9 days (Figure 4and Figure S2). Earlier, the upregulation of ethylene biosynthesis genes was clearly demonstrated during natural and induced senescence in *Medicago truncatula* Gaertn., pea, and *L. luteus* nodules [44,48,58,65]. Obviously, like ABA, ethylene is an essential player in the activation of nodule senescence at the early stages of exposure to elevated temperature. Previously, an increase in the ACC level was observed with the aging of pea nodules during natural senescence. At the same time, in inefficient pea mutants, the level of ACC was significantly higher than in nodules of corresponding wild types of the same age. It should be noted that a high intensity of the ACC label was observed in degraded cells both in natural and induced senescence [48,58]. In addition, immunolocalization revealed a high level of ACC in *L. luteus* nodules exposed to drought stress [65]. It seems that ethylene controls not only the primary responses to heat stress in nodules, but also the stage of deep degradation of nodule symbiotic structures. The involvement of ethylene in the regulation of heat stress-activated pea symbiotic nodule senescence was also confirmed using the ethylene synthesis inhibitor AVG, which delayed nodule senescence to a certain extent (Figure 8B,F and Figure S5B,F).

In heat-stressed nodules, a gradual decrease in the amount of the GA_3_ label was observed during aging (Figure 5Figure 6, and Figure S4). Previously, a decline in the GA_3_ label was clearly demonstrated during natural and induced senescence in pea nodules [49]. It should be noted that a decrease in the amount of gibberellins and repression of their signaling is a typical response to various stresses in a plant [66]. Indeed, in *Arabidopsis thaliana* (L.) Heynh. under cold stress, activation of *GA2ox* and *GA2ox7* gene expression and repression of *GA20ox* gene were observed, leading to a decrease in GA content [67,68,69]. Also, inhibition of *GA20ox* and *GA3ox* gene expression and low GA content was shown during *A. thaliana* seed development when exposed to high temperature [70].

At the same time, exogenous GA_3_ was shown to promote nitrogen fixation and reduce senescence zones in pea wild-type nodules [49]. Those findings strongly indicated the negative effect of gibberellins on nodule senescence activated at the basal part. In this study, GA_3_ treatment reduced expression levels of genes associated with senescence, heat shock, and defense responses (Figure 9) and promoted the nitrogen fixation zone in heat-stressed nodules (Figure 7B,D and Figure 8B,D). It is interesting to note that foliar treatment with GA_3_ was able to mitigate heat stress in tomato (*Solanum lycopersicum* L.) plants [71]. Also, exogenous GA_3_ reduced the effect of salt stress in soybean (*Glycine max* L. (Merill)) [72].

Thus, this study clearly demonstrated that apical senescence in heat-stressed nodules is controlled by phytohormones in the same mode as senescence in the basal part of the nodule activated with aging and other stressors. ABA and ethylene promote senescence, while gibberellins are negative regulators. Further research should be aimed at studying the possibility of practical application of treating pea crops with gibberellic acid to reduce the negative effects of high temperatures.

## 4. Materials and Methods

### 4.1. Plant Material, Bacterial Strain and Plant Growth Conditions

The pea (*Pisum sativum* L.) laboratory line SGE [73] was used in this study. The procedure for seed sterilization was described earlier [74]. Seedlings were inoculated with *Rhizobium leguminosarum* bv. *viciae* 3841 strain [75]. Plants were grown in a growth chamber MLR-352H (Sanyo Electric Co., Ltd., Moriguchi, Japan) under controlled conditions (day/night, 16/8 h; temperature, 21 °C; relative humidity, 75%; photosynthetic photon flux density of ~280 μmol photons m^−2^ s^−1^ for 21 days after inoculation) with the following change of growth conditions in accordance with the design of the experiment.

### 4.2. Growing of Plants at Elevated Temperature

Twenty-one days after inoculation, half of the plants were transferred to another MLR-352H growth chamber with an elevated temperature of 28 °C (heat-stressed plants). Another half of the plants, which continued to grow at 21 °C, were used as a control (heat-unstressed plants). Collection of material for the immunolocalization of phytohormones was carried out in 1 (22-day-old plants), 5 (26-day-old plants), and 9 (30-day-old plants) days after the beginning of exposure to elevated temperature (Figure S6).

### 4.3. Cultivation of Plants of Pea Line SGE under Elevated Temperature Conditions in Combination with Pharmacological Treatment

In the second type of experiment, pharmacological treatment with aqueous solutions of 1 μM gibberellic acid (GA_3_) (Sigma-Aldrich, St Louis, MO, USA) or 100 μM aminoethoxyvinylglycine hydrochloride (AVG, ethylene biosynthesis inhibitor) (Sigma-Aldrich) was started simultaneously with the transfer of the group of plants to an elevated temperature. The addition of 100 mL of a solution (GA_3_ or AVG) to the substrate was carried out with an interval of 2–3 days to the groups of the plants grown at 21 and 28 °C. Plants grown at 21 and 28 °C without treatment and those grown at 21 °C treated with GA_3_ or AVG solutions were used as controls. In each variant, 15–20 plants were grown. Each of the three biological replicates included 5–7 plants. Nodules were harvested for the analysis of their phenotype, histological structure, and expression analysis in 7 and 9 days after the beginning of exposure at 28 °C and pharmacological treatment (Figure S7).

### 4.4. Nodule Phenotypes

Photographs of plant roots with symbiotic nodules were taken using a stereomicroscope SteREO Lumar.V12 (Carl Zeiss, Oberkochen, Germany) equipped with AxioCam MRc 5 camera (Carl Zeiss) and the AxioVision Rel. 4.8 software (Carl Zeiss). The nodule phenotypes were analyzed in 4–6 plants within each variant.

### 4.5. Light Microscopy

For histological structure analysis, nodules were fixed, embedded in Steedman’s wax, and stained with toluidine blue according to the earlier described protocol [48]. Light microscopy analysis of 10 µm longitudinal pea nodule sections was carried out with AxioImagerZ1 (Carl Zeiss). Images were taken with a microscope camera AxioCam 506 color (Carl Zeiss) and processed using the ZEN 2 Core SP1 software V 2.0 (Carl Zeiss). For each variant, sections of 7–10 nodules from 6–10 plants were analyzed.

### 4.6. Immunolocalization of Phytohormones

Experiments for immunolocalization of phytohormones were repeated five times. Each variant consisted of 10 plants in 2 pots (5 plants in each pot). Immunolabeling of ACC and GA_3_ was conducted as previously described [48,49]. Abscisic acid (ABA) was visualized following the technique of Peng and co-authors with modifications [76]. For fixation of ABA, nodules were incubated in a 4% solution of N-(-3-dimethylaminopropyl)-N′-ethylcarbodiimide hydrochloride (Sigma-Aldrich) in 1/4 MTSB buffer (50 mM PIPES, 5 mM MgSO_4_·7H_2_O, 5 mM EGTA, pH 6.9) with 0.1% Tween-20 and 0.1% Triton X-100 under vacuum (−0.9 bar) infiltration during 30 min using a VacuuBrand ME 1C vacuum pump (Vacuubrand, Wertheim, Germany). Then nodules were fixed in a solution of 4% paraformaldehyde in 1/4 MTSB with 0.1% Tween-20 and 0.1% Triton X-100 under vacuum (−0.9 bar; air from tissues was pumped out for 15 min; then, for 6 min, nodules were incubated with air; the procedure was repeated 5 times) and were left at 4 °C overnight. After that, nodules were washed 4 times for 15 min in a buffer and used for fixative solution. Then nodules were molded in 3% agarose gel blocks. Longitudinal sections with a thickness of 50 μm were prepared using a microtome with a vibrating blade HM650V (Microm International GmbH, Walldorf, Germany). Immunolocalization of ABA was performed using primary antibodies to ABA (Agrisera, Vännäs, Sweden) in a dilution 1:100. Goat anti-rabbit antibodies conjugated with Alexa Fluor 488 (Thermo Fisher Scientific, Waltham, MA, USA) in a dilution 1:750 were used as secondary antibodies. Nuclei and bacteria were stained with propidium iodide (0.5 µg ml^−1^) for 7 min, then washed in TBS buffer 2 times and mounted in ProLong Diamond antifade reagent (Thermo Fisher Scientific). Slides were examined using the laser scanning confocal microscope LSM 510 META (Carl Zeiss) and ZEN 2012 software (Carl Zeiss). AlexaFluor 488 was excited at 488 nm, and fluorescence emitted between 499 to 543 nm was collected; propidium iodide was excited at 561, and emitted fluorescence between 606 and 677 nm was collected.

### 4.7. Expression Analysis of Genes Associated with Senescence, Heat Stress, and Defense Responses

Primers for genes associated with (i) senescence (cysteine protease 15a (*PsCyp15a*), P45 subunit of the AAA-ATPase 26S proteasome (*Ps26S AAA-ATPase*), transcription factor bZIP (*PsATB2*), enzymes for GA metabolism (*PsGA20ox1*, *PsGA2ox1*), ethylene biosynthesis (*PsACS2*, *PsACO1*), and ABA biosynthesis (*PsNCED2*, *PsAO3*)); (ii) heat stress (heat shock proteins (*PsHSP22*, *PsHSP17.9*, *PsHSP70*)); and (iii) defense responses (biosynthesis of jasmonates (*PsLoxN1*) and glutathione (*PsGSH1*, *PsGSHS*), marker of hypersensitivity reaction (*PsHSR203J*), and disease resistance response protein (*PsPR10*)) [40,48,74,77,78,79,80] were designed with the VectorNTI Advanced 10 software version 11.5.1 (Invitrogen, Carlsbad, CA, USA) and were synthesized by Evrogen (Moscow, Russia) (Table S2). Total RNA was isolated using the TRIzol Reagent (Ambion Inc., Austin, TX, USA) according to the manufacturer’s recommendations. RNA quantification was carried out using Qubit 2.0 (Invitrogen). cDNA synthesis on total RNA (2.5 µg) and relative real-time PCR were performed as described previously [49]. The relative expression was calculated with the ΔCt method using the reference gene *PsGapC1* gene (L07500.1, Table S2). Statistical treatment of data was processed using the Microsoft Excel 2016 software. The experiments were performed in three biological replicates with five to six plants per variant. Statistically significant differences were calculated using one-way ANOVA followed by a *t*-test with multiple testing correction; a *p*-value < 0.05 was considered to be significant. Relative expression levels were visualized using the pheatmap package version 1.0.12 [81].

## Data Availability

Data generated for this study are included in the manuscript and/or the Supplementary Material.

## Supplementary Materials

The following supporting information can be downloaded at https://www.mdpi.com/article/10.3390/ijms242317062/s1.
