# Supplementary material for: Effects of Elevated Temperature on Pisum sativum Nodule Development: II—Phytohormonal Responses
Source: Int J Mol Sci. 2023 Dec 2;24(23):17062. doi: 10.3390/ijms242317062 (PMC10707278; doi:10.3390/ijms242317062)
Supplement: Supplementary file 1 [file ijms-24-17062-s001.zip › Kitaeva_2023_supplementary_data.pdf]

SUPPLEMENTARY DATA for

Effects of elevated temperature on *Pisum sativum* nodule development: II—Phytohormonal responses

Anna B. Kitaeva*,†, Tatiana A. Serova†, Pyotr G. Kusakin, and Viktor E. Tsyganov*

Laboratory of Molecular and Cell Biology, All-Russia Research Institute for Agricultural Microbiology, Podbelsky Chaussee 3, 196608, Pushkin 8, Saint Petersburg, Russia

* Authors for correspondence: Anna B. Kitaeva (akitaeva@arriam.ru), Viktor E. Tsyganov (vetsyganov@arriam.ru)

† These authors contributed equally to this work.

Figure S1. Immunolocalization of abscisic acid (ABA) in 30-day-old nodules of the pea (*Pisum sativum*) line SGE. (**A–F**) Heat-unstressed nodules, (**G–L**) heat-stressed nodules. (**A–C**,**G–I**) Whole nodules; (**D–F**) nitrogen fixation zone; (**J–L**) senescence zone. Confocal laser scanning microscopy of 50 μm longitudinal vibratome sections. (**A,D,G,J**) Merge of differential interference contrast and red channel (DNA staining with propidium iodide (nuclei and bacteria)). (**B,E,H,K**) Merge of green (ABA) and red (propidium iodide) channels. (**C,F,I,L**) The heatmap shows color-coded fluorescence signal intensities for the green signal channel; the quantification scale is the same for all images. I, meristem; II, infection zone; III, nitrogen fixation zone; IV, senescence zone. ic, infected cell; uic, uninfected cell; dic, degraded infected cell; duic, degraded uninfected cell; n, nucleus; arrows indicate infection threads. Scale bars are 100 μm (**A–C**,**G–I**) and 20 μm (**D–F**,**J–L**).

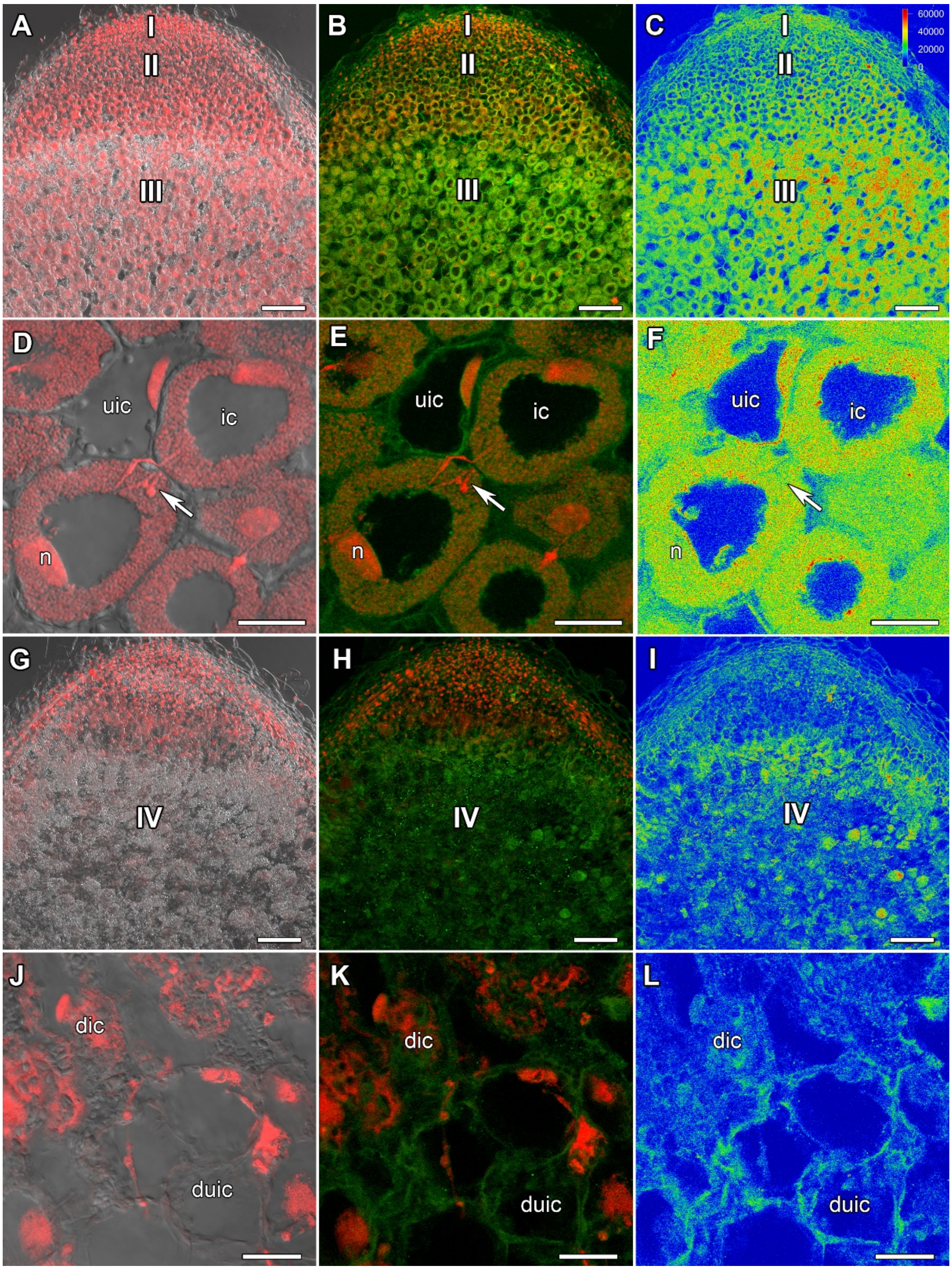

Figure S2. Immunolocalization of 1-aminocyclopropane-1-carboxylate (ACC) in 30-day-old nodules of the pea (*Pisum sativum*) line SGE. (**A–F**) heat-unstressed nodules, (**G–L**) heat-stressed nodules. (**A–C**,**G–I**) Whole nodules; (**D–F**) nitrogen fixation zone; (**J–L**) senescence zone. Confocal laser scanning microscopy of 50 μm longitudinal vibratome sections. (**A**,**D**,**G**,**J**) Merge of differential interference contrast and red channel (DNA staining with propidium iodide (nuclei and bacteria)). (**B**,**E**,**H**,**K**) Merge of green (ACC) and red (propidium iodide) channels. (**C**,**F**,**I**,**L**) The heatmap shows color-coded fluorescence signal intensities for the green signal channel; the quantification scale is the same for all images. I, meristem; II, infection zone; III, nitrogen fixation zone; IV, senescence zone. ic, infected cell; uic, uninfected cell; dic, degraded infected cell; duic, degraded uninfected cell; n, nucleus. Scale bars are 100 μm (**A–C**,**G–I**), 10 μm (D–F) and 20 μm (**J–L**).

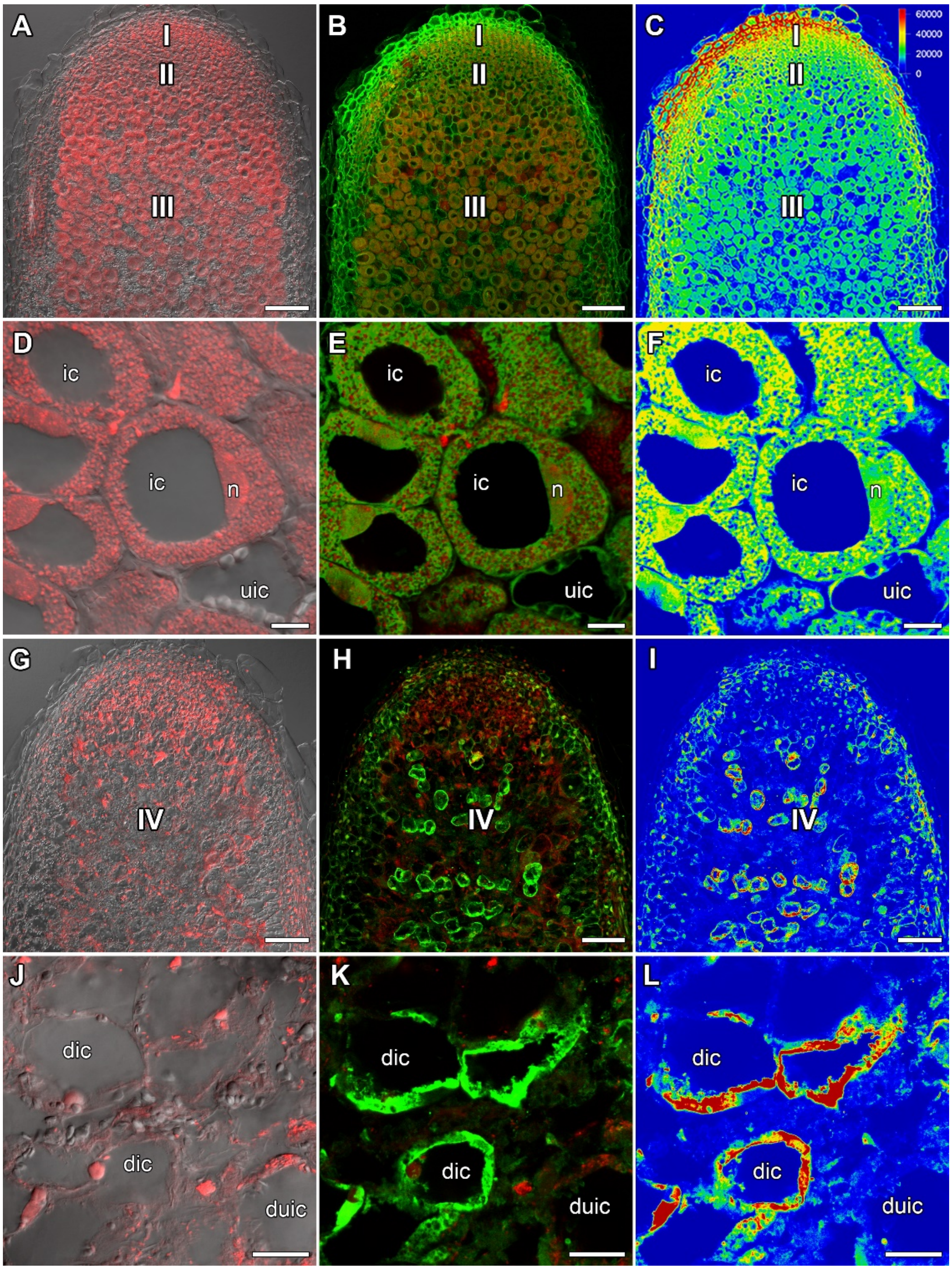

Figure S3. Immunolocalization of gibberellic acid (GA_3) in 30-day-old nodules of the pea (*Pisum sativum*) line SGE. (**A–F**) heat-unstressed nodules, (**G–L**) heat-stressed nodules. (**A**–**C**,**G**–**I**) Whole nodules; (**D**–**F**) nitrogen fixation zone; (**J**–**L**) senescence zone. Confocal laser scanning microscopy of 50 μm longitudinal vibratome sections. (**A,D,G,J**) Merge of differential interference contrast and red channel (DNA staining with propidium iodide (nuclei and bacteria)). (**B,E,H,K**) Merge of green (GA_3) and red (propidium iodide) channels. (**C,F,I,L**) The heatmap shows color-coded fluorescence signal intensities for the green signal channel; the quantification scale is the same for all images. I, meristem; II, infection zone; III, nitrogen fixation zone; IV, senescence zone. ic, infected cell; uic, uninfected cell; dic, degraded infected cell; duic, degraded uninfected cell; n, nucleus. Scale bars are 100 μm (**A–C**,**G–I**) and 20 μm (**D–F**,**J–L**).

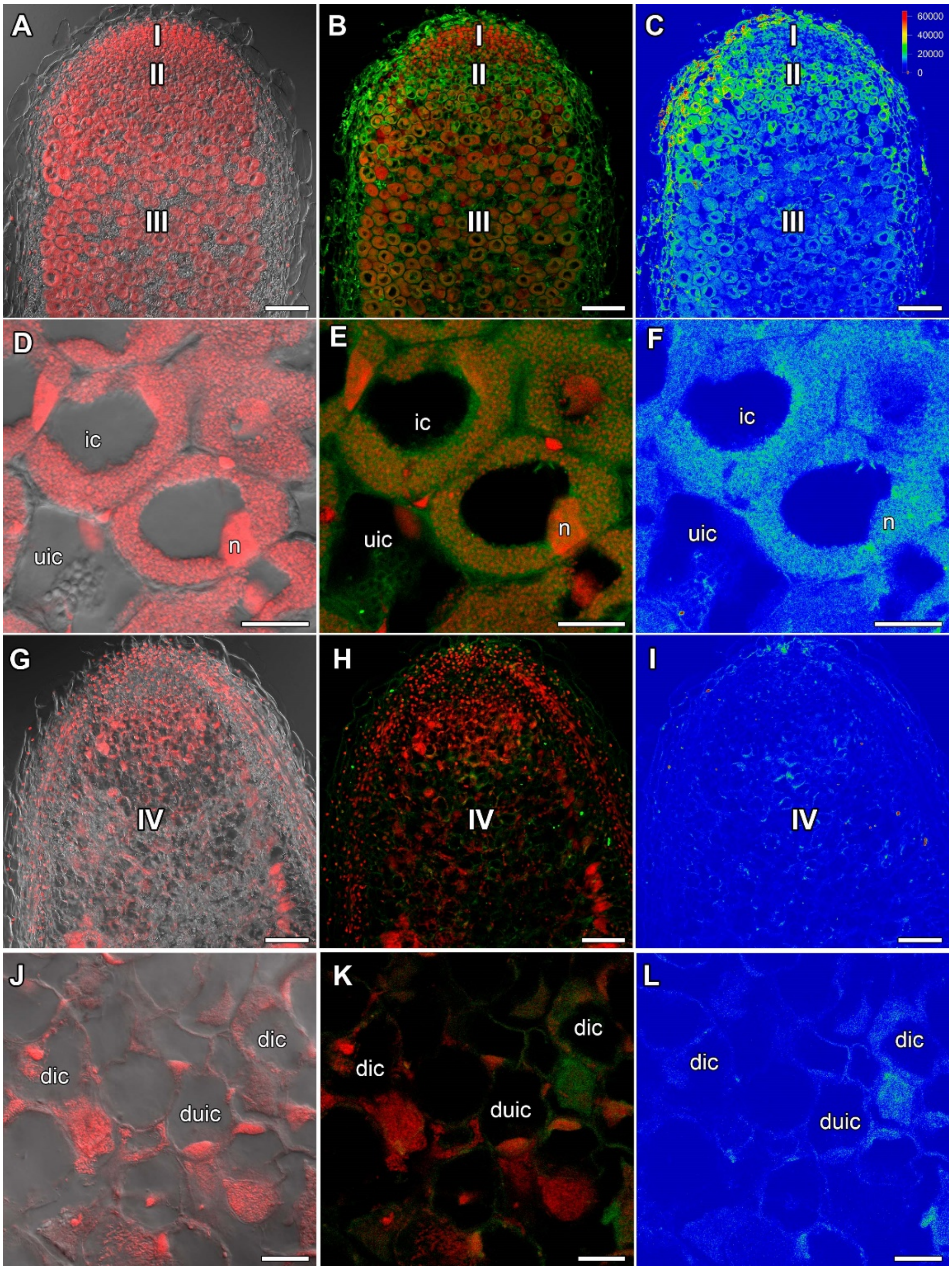

Figure S4. Heat-unstressed (A, C, E) and heat-stressed (B, D, F) 30-day-old nodules of the pea (*Pisum sativum*) line SGE untreated (A, B) and treated with 1 μM gibberellic acid (GA_3) (C, D) or 100 μM aminoethoxyvinylglycine hydrochloride (AVG) (E, F). Scale bars are 2 mm.

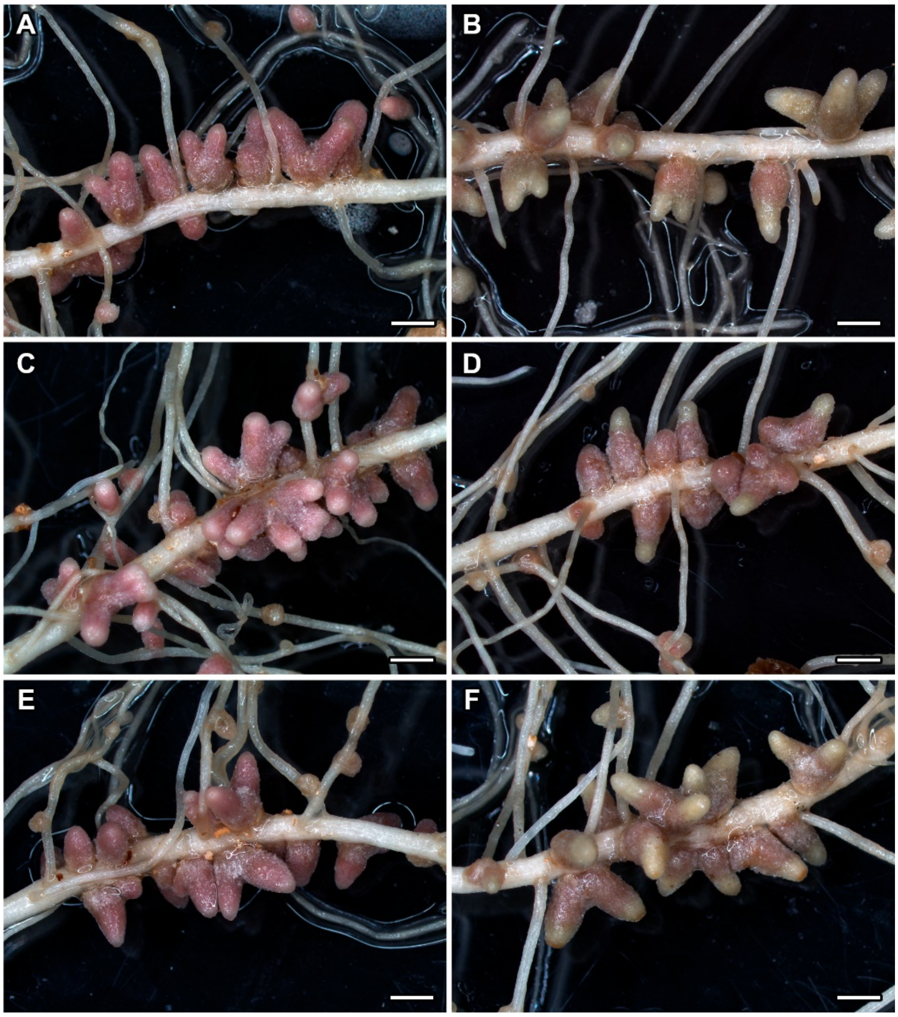

Figure S5. Histological organization of heat-unstressed (A, C, E) and heat-stressed (B, D, F) 30-day-old nodules of the pea (*Pisum sativum*) line SGE untreated (A, B) and treated with 1 μM gibberellic acid (GA_3) (C, D) or 100 μM aminoethoxyvinylglycine hydrochloride (AVG) (E, F). Light microscopy of 10 μm longitudinal microtome sections, stained with toluidine blue. I, meristem; II, infection zone; III, nitrogen fixation zone; IV, senescence zone; IV*, senescence zone at the apical part of the nodule. Scale bars are 500 μm.

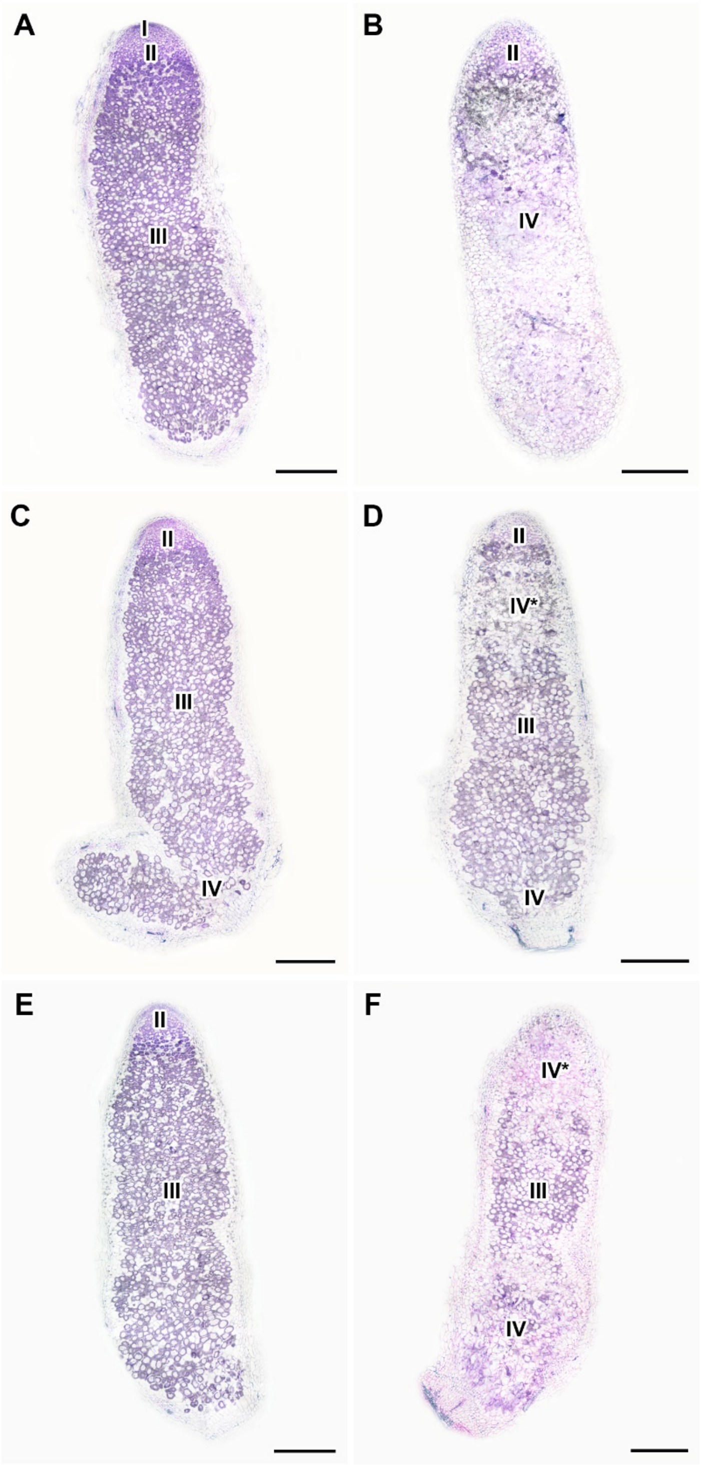

Figure S6. Scheme of the experiment on the effect of exposure to elevated temperature (28 °C) on the localization of abscisic acid, 1-aminocyclopropane-1-carboxylic acid, and gibberellic acid in pea (*Pisum sativum*) line SGE nodules. DAI, days after inoculation with *Rhizobium leguminosarum* bv. *viciae* 3841.

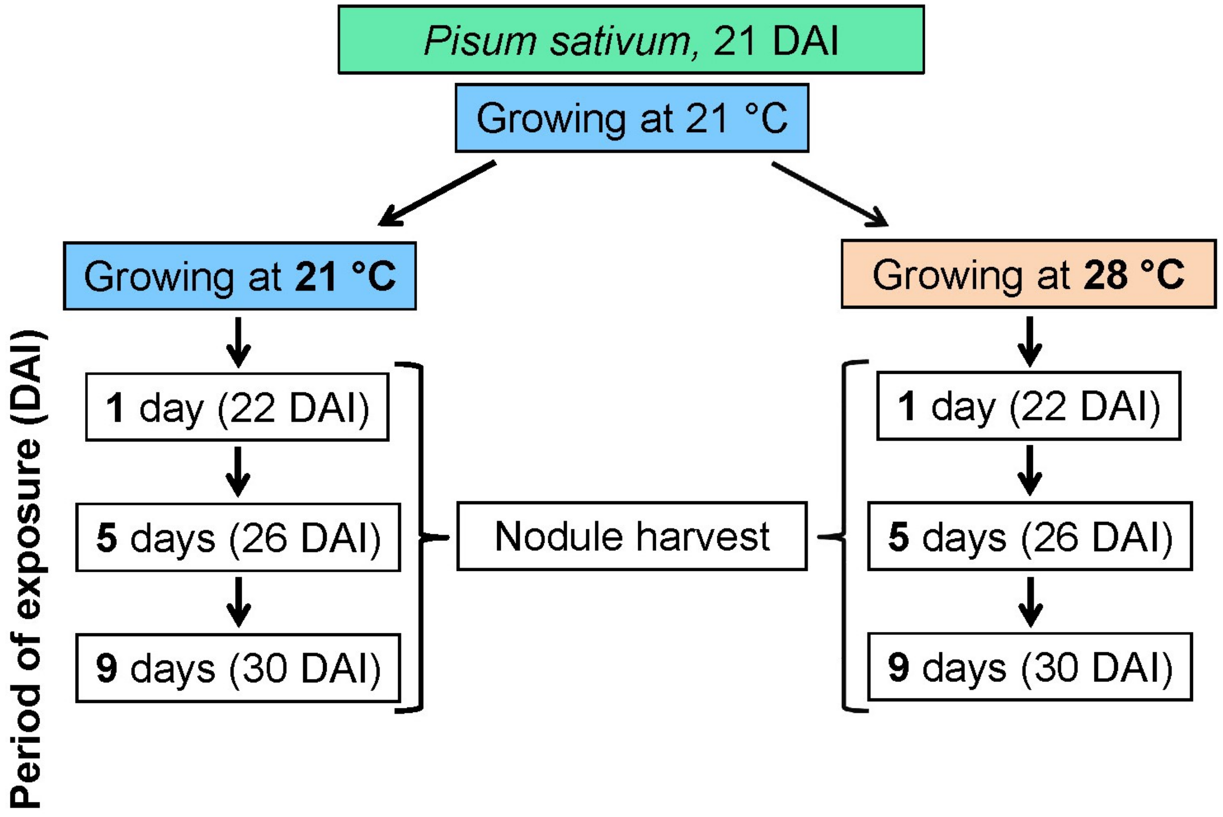

Figure S7. Scheme of the experiment on the effect of exposure to elevated temperature (28 °C) in combination with pharmacological treatment [1 μM gibberellic acid (GA_3) or 100 μM aminoethoxyvinylglycine hydrochloride (AVG)] on the functioning of pea (*Pisum sativum*) nodules. DAI, days after inoculation with *Rhizobium leguminosarum* bv. *viciae* 3841.

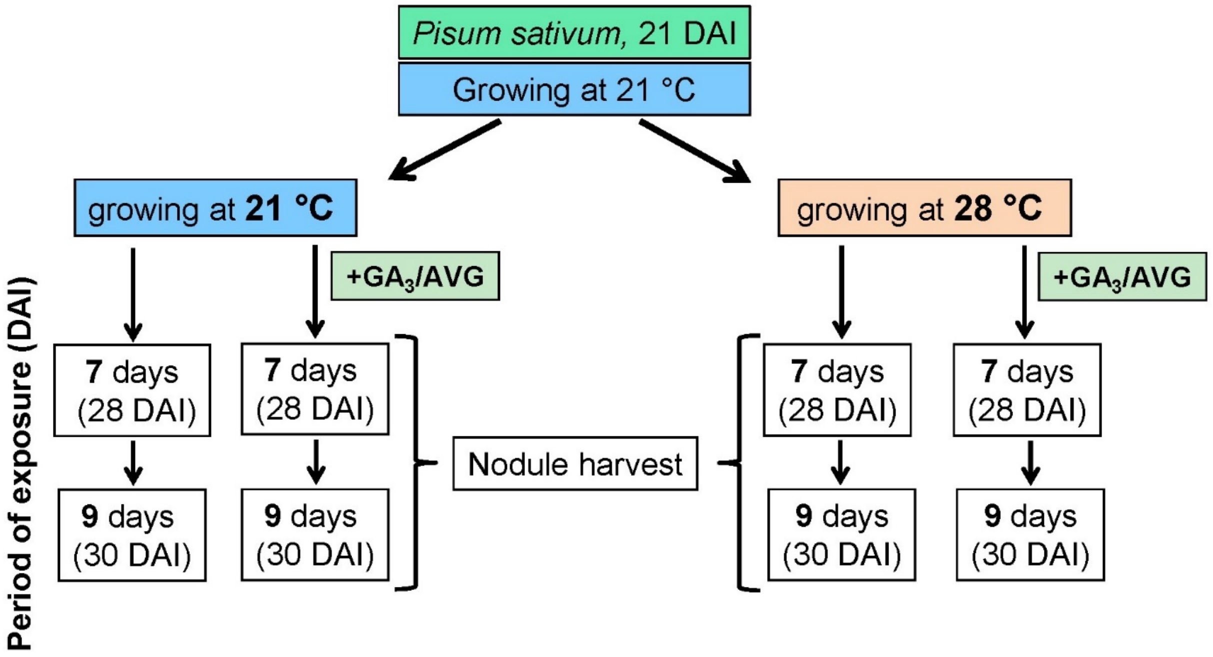

Table S1. The relative expression levels of genes in nodules of pea (*Pisum sativum*) plants of the SGE line exposed to 21 °C or 28 °C for 7 or 9 days with or without gibberellic acid (GA3) treatment (list 1) and statistical analysis of gene expression (list 2).

Table S2. Description of primer sequences for expression analysis of *Pisum sativum* selected genes.

**Gene**	**Description**	**Accession no.**	**Primer sequence (5′–3′)**	**Amplicon size (bp)**	**Reference**
PsCyp15a	Cysteine protease 15a	X54358.1	^{1107}GTAGCTGCAGCTCAATCCAACC<br>^{1304}CATCACCACAGTAACAGCAAGACA	222	[48]
Ps26S AAA-ATPase* ***	26S proteasome AAA-ATPase subunit	PsCam000968*	^{8} AATGGGGTCACGTAACATAGCG<br>^{105} GATGGAAGGGGTGAAGGTTAGG	119	[40]
PsATB2	bZIP transcription factor	69046**	^{104}GAGACGGTCTCGGATGAGGAAA<br>^{351}TCAGAGGGTTGAAGAAGAAGAAGC	272	[48]

PsHsr203J	Hypersensitivity response marker	AB026296.1	^{175}CACTACCACCAACGACAACTTCA<br>^{214}GGCGTTTTCTCCGGTAGGTAT	60	[74, 77]
PsACS2	ACC synthase 2	AF016459.1	^{1495}GGCATAGTAATTTGAGGTTGAGCC<br>^{1695}GCCCCAACATTTAAAGGACCTATTA	226	[48]
PsACO1	ACC oxidase 1	M98357.1	^{862}TACATGGGACTCAAGTTCCAAGCT<br>^{995}GCACAATCTTAAAACACCAACCAAA	159	[48]
PsNCED2	9-cis-epoxycarotenoid dioxygenase 2	AB080192.1	^{96}GAACCAATCTTCTCCACTATGGCA<br>^{232}AAGGGAGTGTTGTTTGTAGCGAAC	163	[40]
PsAO3	Aldehyde oxidase 3	EF491600.1	^{4386}TTATAGGACACAGGCTAGCTCAGCA<br>^{4487}TGACACAAGCTTATTCAGCATGACA	127	[48]
PsGA20ox1	GA 20-oxidase 1	U70471.1	^{874}CATTCCATTAGGCCAAATTTCAAT<br>^{945}CTGCCCTATGTAAACAACTCTTGTATCT	100	[78]

PsGA2ox1	GA 2-β-hydroxilase 1	AF056935.1	[1009]GCTGCCACTTAATATTGGAGGATC<br>[1236]GAGTGTTGATGCAAAAGGGGAA	250	[48]
PsLoxN1	Lipoxygenase 1	U84198.1	[71]ACATGGCAACAAAGGTGTTTGG<br>[162]TTTACCGATGGACGTTATAGCG	113	[40]
PsHSP70	Chloroplastic heat shock protein 70 kDa	L03299.1	[36]CTCTTCACTCATGGCTTCTTCTGC<br>[128]TGACCGAAGAAAAGGGTTTTGG	114	This study
PsHSP22	Heat shock protein 22 kDa	X86222	[35]TCTCTCCTTCAAATACAACCGCC<br>[121]GAAGCCATTGAGATTGCGGG	106	[40]
PsHSP17.9	Heat shock protein 17.9 kDa	M33900	[20]GGTACTGGACGAAGAACCAATGC<br>[117]AAAAGCTGCCGTCTCGTTGG	117	[40]

PsGSH1	γ-glutamylcysteine synthetase	AF128455.1	203CTCCTCCGCCGCATAACTTC<br>373GGCGAGATAATCGATGAGATCCTG	194	[79]
PsGSHS	Glutathione synthetase	AF231137.1	28GCCGCTGATTTTCGTTCCACTA<br>193CGACGTCGACGGTTTGTTTACC	187	[79]
PsPR10	Disease resistance response protein PR10 (DRR49a), putative RNAse	U31669.1	1335GCCGGAACCATCAAGAAACT<br>1680GCCTTGAAAAGACCATCACCC	366	[79]
PsGapC1	Glyceraldehyde-3-phosphate dehydrogenase	L07500.1	222AAGAACGACGAACTCACCG<br>389TTGGCACCACCCTTCAAATG	188	[80]

* contig sequence for *P. sativum* in the database https://urgi.versailles.inra.fr/

** contig sequence for *P. sativum* in the database https://www.coolseasonfoodlegume.org/

*** gene symbols were created by the authors based on the annotation of contig sequence
